# Risk factors for thromboembolic complications in isolated severe head injury

**DOI:** 10.1007/s00068-023-02292-y

**Published:** 2023-06-08

**Authors:** Dominik A. Jakob, Martin Müller, Meghan Lewis, Monica D. Wong, Aristomenis K. Exadaktylos, Demetrios Demetriades

**Affiliations:** 1 Division of Trauma and Surgical Critical Care, Department of Surgery, Los Angeles County, University of Southern California Medical Center, University of Southern California, Los Angeles, CA 90033 USA; 2 Department of Emergency Medicine, Inselspital, Bern University Hospital, University of Bern, 3010 Bern, Switzerland

**Keywords:** Head trauma, Mechanism of injury, Pulmonary embolism, Deep vein thrombosis

## Abstract

**Purpose:**

Patients with traumatic brain injury (TBI) are at high risk for venous thromboembolism (VTE). The aim of the present study is to identify factors independently associated with VTE events. Specifically, we hypothesized that the mechanism of penetrating head trauma might be an independent factor associated with increased VTE events when compared with blunt head trauma.

**Methods:**

The ACS-TQIP database (2013–2019) was queried for all patients with isolated severe head injuries (AIS 3–5) who received VTE prophylaxis with either unfractionated heparin or low-molecular-weight heparin. Transfers, patients who died within 72 h and those with a hospital length of stay < 48 h were excluded. Multivariable analysis was used as the primary analysis to identify independent risk factors for VTE in isolated severe TBI.

**Results:**

A total of 75,570 patients were included in the study, 71,593 (94.7%) with blunt and 3977 (5.3%) with penetrating isolated TBI. Penetrating trauma mechanism (OR 1.49, CI 95% 1.26–1.77), increasing age (age 16–45: reference; age > 45–65: OR 1.65, CI 95% 1.48–1.85; age > 65–75: OR 1.71, CI 95% 1.45–2.02; age > 75: OR 1.73, CI 95% 1.44–2.07), male gender (OR 1.53, CI 95% 1.36–1.72), obesity (OR 1.35, CI 95% 1.22–1.51), tachycardia (OR 1.31, CI 95% 1.13–1.51), increasing head AIS (AIS 3: reference; AIS 4: OR 1.52, CI 95% 1.35–1.72; AIS 5: OR 1.76, CI 95% 1.54–2.01), associated moderate injuries (AIS = 2) of the abdomen (OR 1.31, CI 95% 1.04–1.66), spine (OR 1.35, CI 95% 1.19–1.53), upper extremity (OR 1.16, CI 95% 1.02–1.31), lower extremity (OR 1.46, CI 95% 1.26–1.68), craniectomy/craniotomy or ICP monitoring (OR 2.96, CI 95% 2.65–3.31) and pre-existing hypertension (OR 1.18, CI 95% 1.05–1.32) were identified as independent risk factors for VTE complications in isolated severe head injury. Increasing GCS (OR 0.93, CI 95% 0.92–0.94), early VTE prophylaxis (OR 0.48, CI 95% 0.39–0.60) and LMWH compared to heparin (OR 0.74, CI 95% 0.68–0.82) were identified as protective factors for VTE complications.

**Conclusion:**

The identified factors independently associated with VTE events in isolated severe TBI need to be considered in VTE prevention measures. In penetrating TBI, an even more aggressive VTE prophylaxis management may be justified as compared to that in blunt.

**Supplementary Information:**

The online version contains supplementary material available at 10.1007/s00068-023-02292-y.

## Introduction

According to the Centers for Disease Control and Prevention (CDC) traumatic brain injury (TBI) remains a major health problem. In 2020 alone, 64,000 TBI-related deaths were recorded in the United States, which corresponds to 176 TBI-related death per day [1]. Approximately 3–4 times as many patients are hospitalized each year for TBI in the United States [1]. The primary goal of TBI management must focus on preventing secondary injury by avoiding hypotension and hypoxia. However, other important factors in the complex management of TBI that need to be considered are venous thromboembolism (VTE) prevention, metabolic and nutritional optimization, as well as seizure and stress ulcer prophylaxis [2].

All trauma patients are at high risk for VTE [3–6], and especially those who sustain TBI have an even higher risk for VTE [7, 8]. Without the administration of VTE prophylaxis, VTE rates in TBI are reported as high as 53.8% [3, 9]. The high VTE rates in TBI may be explained by procoagulants, namely tissue factors that are rich in the brain tissue. Following trauma a systematic release may trigger a hypercoagulable state [10]. Other factors, such as long immobilization periods, including TBI related focal motor deficit, and the frequent need for neurosurgical procedures, may also contribute to the high VTE risk in this subset of patients. There are limited data available on risk factors for VTE events in TBI. In particular, it is unclear how the mechanism of injury alters the risk for VTE. Martin et al. [11] showed that patients with penetrating TBI demonstrates increased coagulopathy [measured by thromboelastography (TEG) and need for transfusion] compared to those with blunt TBI. However, the role of the mechanism of injury regarding the development of VTE events has not been determined in TBI.

The current study was initiated to evaluate factors associated with VTE events after isolated severe TBI. Specifically, we hypothesized that penetrating TBI is independently associated with increased VTE complications compared to blunt TBI. Our findings may help for improved characterization of VTE risk factors and will help to identify high risk population that merit more aggressive VTE prophylaxis, monitoring and screening.

## Methods

### Study design

This is a retrospective observational cohort study using the American College of Surgeons (ACS) Trauma Quality Improvement Program (TQIP) database from January 2013 to December 2019. The TQIP database is maintained by the American College of Surgeons Committee on Trauma and aggregates patient data from more than 760 trauma centers across the United States [12].

### Search strategy and eligibility criteria

**Fig. 1:**
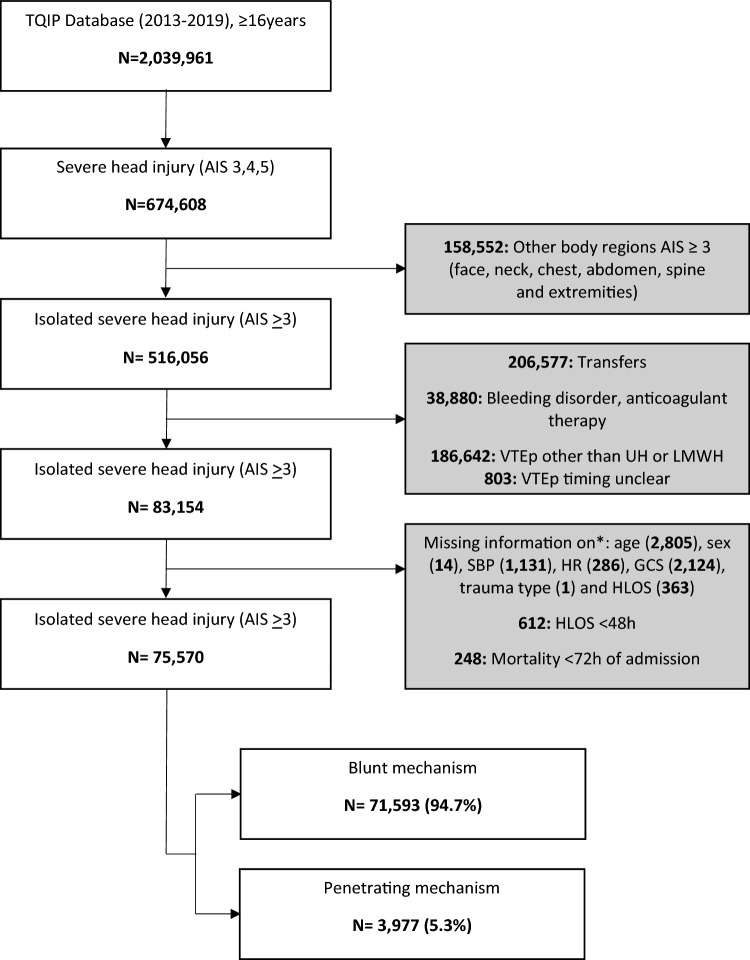
Patient flowchart. *TQIP* trauma quality and improvement program; *AIS* abbreviated injury scale; *VTEp* venous thromboembolism prophylaxis; *UH* unfractionated heparin; *LMWH* low molecular weight heparin; *SBP* systolic blood pressure; *HR* heart rate; *GCS* Glasgow coma scale; *HLOS* hospital length of stay; *h* hours

The database was queried to identify all patients (≥ 16 years old) who sustained severe TBI, defined as head abbreviated injury scale (AIS) 3–5. Patients with isolated severe head injury were then extracted by excluding those with face, neck, chest, abdomen, spine, extremity and external/other/unspecified AIS > 2. Other exclusion criteria were transfers, bleeding disorders or anticoagulant therapy, pharmacological venous thromboembolic other than unfractionated heparin (UH) or low molecular weight heparin (LMWH), death within 72 h of admission, hospital length of stay < 48 h. In addition, patients with missing information on age, sex, heart rate (HR), systolic blood pressure (SBP), Glasgow Coma Score (GCS), VTE timing, trauma type and hospital length of stay (HLOS) were excluded. The patient flowchart including missing or unspecified data shown in Fig. 1. Owing to the small numbers of missing key variables (all less than 5%), these patients were omitted from analysis and imputation was not performed.

### Data collection and extraction

For all patients with isolated severe TBI the following variables were extracted from the TQIP database:

(i) demographic data, such as age, gender, race, height (cm) and weight (kg),
(ii) documented comorbidities, including admission data [HR, SBP, GCS] and mechanism of injury (blunt vs penetrating),
(iii) data on anatomic location and severity of the injury by calculating the Abbreviated Injury Scale [AIS] for each body region and each patient,
(iv) timing and type of VTE prophylaxis and surgical procedures [craniectomy/craniotomy and intracranial pressure monitoring (ICP) monitoring]. Craniectomies and craniotomies were identified from the TQIP database by extracting all ICD 9 and ICD 10 procedure codes associated with a neurosurgical operation; ICP monitoring data were obtained from the separate variable recorded in the TQIP database.
(v) data on primary outcome parameters included pulmonary embolism (PE), deep venous thrombosis (DVT) summarized as VTE (PE + DVT). In addition, secondary outcome parameters including total hospital length of stay, total ICU length of stay and in-hospital mortality were extracted for each patient.

The definitions for all collected variables can be found in the National Trauma Data Standard provided by the ACS [13]. Hypotension was defined as a blood pressure < 90 mmHg, and tachycardia was defined as a heart rate > 120 beats per minute. Body Mass Index (BMI) was calculated by the patient’s documented weight in kilograms divided by the square of height in meters. Obesity was defined by a BMI above 30 kg/m^2^ according to the World Health Organization recommendations [14].

### Statistical analysis

Normality of distribution was assessed using histograms, skewness, kurtosis, and the Shapiro–Wilk test. In order to test our hypothesis patients were divided into blunt and penetrating injuries based on the mechanism reported. Univariable analysis was used to identify differences in baseline and outcome variables between patients with blunt vs penetrating injuries. Categorical variables were compared using Chi-Square test. Mann–Whitney *U* test was used to compare medians for continuous variables. The results were reported as numbers and percentages for categorical variables or medians and interquartile ranges (IQR) for continuous variables.

Multivariable logistic regression analysis was performed in order to identify independent risk factors for thromboembolic events (DVT + PE), and mortality. Clinically relevant variables with a *p* value < 0.2 on univariate analysis and known risk factors for VTE events were included into the analysis. We used a stepwise backward selection model (*p* < 0.05) in order to identify important variables for our final model. The results were reported as odds ratios (ORs) and 95% confidence intervals (CIs). Correlation between variables were tested with multicollinearity analysis using the—*collin*—command. A Variance Inflation Factor of less than 2.5 was used as a problematic amount of multicollinearity [15]. The area under the receiver operating characteristic curve with 95% CI was used to assess the accuracy of the test. Variables with *p* value < 0.05 were considered significant. Stata/SE® 16.1 (StataCorp, The College Station, Texas, USA) was used for statistical analysis.

### Sensitivity analysis

For sensitivity analysis, an additional 1:4 case control matching of patients with penetrating vs blunt trauma mechanism was performed on the basis of the following criteria: age categories, sex, head AIS, extracranial AIS 2 for face, neck, chest, abdomen, spine, upper and lower extremities. On the basis of the matched cohorts a subsequent regression analysis was performed in order to identify independent predictors for VTE complications.

### Ethical considerations

The study was approved by the Institutional Review Board of the University of Southern California (HS-21-00258-AM001).

## Results

A total of 75,570 patients with isolated severe TBI met the inclusion criteria. Of these, 71,593 patients (94.7%) sustained blunt and 3,977 patients (5.3%) sustained penetrating trauma (Fig. 1).

### Cohort characteristics

Patients with penetrating injury were significantly younger [31 (23–45) vs 52 (33–67) years, *p* < 0.001], more often male (82.8% vs 70.6%, *p* < 0.001) and more likely to be black (40.0% vs 15.3%, *p* < 0.001) compared with blunt trauma patients. Patients who sustained penetrating trauma were more frequently tachycardic (15.1% vs 7.2%, *p* < 0.001) and hypotensive (5.7% vs 1.3%, *p* < 0.001), and also had a lower GCS on admission [14 (5–15) vs 14 (10–15), *p* < 0.001]. The overall comorbidity rate was higher in blunt trauma patients (58.4% vs 43.1%, *p* < 0.001).

Furthermore, patients with penetrating trauma were more likely to sustain more severe head, face and neck injuries (*p* < 0.001 for AIS head, face and neck), whereas blunt trauma was associated with more severe spine, chest, abdomen, extremity and external injuries when compared with penetrating trauma (*p* < 0.001 for all body areas mentioned).

In penetrating trauma, the utilization of LMWH was 62.5% as compared to 60% in blunt trauma (*p* = 0.002). VTE prophylaxis within 48 h was initiated in 15.7% of penetrating TBI as compared to 13.7% in blunt TBI. Neurosurgical interventions such as craniectomy/craniotomy or ICP monitoring were significantly more common in the penetrating group than in the blunt group. (34.6% vs 15.9%, *p* < 0.001).

DVT (3.5% vs 2.1%, *p* < 0.001) and PE (1.2% vs 0.6%, *p* < 0.001) were significantly more often in penetrating trauma. In hospital mortality was 6.2% in penetrating and 3.7% in blunt trauma, *p* < 0.001 (Table 1).

**Table 1:** Patients’ characteristics, clinical data and outcomes of patients with isolated severe head injury

	Total (*n* = 75,570)		Blunt (*n* = 71,593)		Penetrating (*n* = 3,977)		*p* value
	*n*	*%*	*n*	*%*	*n*	*%*	
Demographics							
Age^a^	51	[32–67]	52	[33–67]	31	[23–45]	< 0.001
Age groups							
16–45	31,803	[42.1]	28,813	[40.2]	2990	[75.2]	< 0.001
> 45–65	23,751	[31.4]	22,955	[32.1]	796	[20.0]	
> 65–75	9425	[12.5]	9289	[13.0]	136	[3.4]	
> 75	10,591	[14.0]	10,536	[14.7]	55	[1.4]	
Gender, male	53,821	[71.2]	50,527	[70.6]	3294	[82.8]	< 0.001
Obesity, BMI > 30 kg/m^2^	15,200	[20.1]	14,289	[20.0]	911	[22.9]	< 0.001
Race							
White	50,169	[66.4]	48,357	[67.5]	1812	[45.6]	< 0.001
Black	12,552	[16.6]	10,963	[15.3]	1589	[40.0]	
Asian	2528	[3.3]	2472	[3.5]	56	[1.4]	
Other^b^	10,321	[13.7]	9801	[13.7]	520	[13.1]	
Vitals							
HR [/min]^a^	88	[75–102]	88	[75–101]	91	[74–110]	< 0.001
Tachycardia (HR > 120 bpm)	5778	[7.6]	5177	[7.2]	601	[15.1]	< 0.001
SBP [mmHg] ^a^	141	[126–160]	142	[126–160]	134	[117–150]	< 0.001
Hypotension (SBP < 90 mmHg)	1134	[1.5]	908	[1.3]	226	[5.7]	< 0.001
GCS^a^	14	[10–15]	14	[10–15]	14	[5–15]	< 0.001
Comorbidites							
Steroid use	366	[0.5]	363	[0.5]	3	[0.1]	< 0.001
Current smoker	12,577	[16.6]	11,761	[16.4]	816	[20.5]	< 0.001
Diabetes mellitus	9431	[12.5]	9263	[12.9]	168	[4.2]	< 0.001
Hypertension	22,868	[30.3]	22,408	[31.3]	460	[11.6]	< 0.001
Cerebrovascular insult	1740	[2.3]	1728	[2.4]	12	[0.3]	< 0.001
Respiratory disease	3385	[4.5]	3281	[4.6]	104	[2.6]	< 0.001
Congestive heart failure	3385	[4.5]	1545	[2.2]	11	[0.3]	< 0.001
Myocardial infarction	504	[0.7]	496	[0.7]	8	[0.2]	< 0.001
Liver cirrhosis	941	[1.2]	928	[1.3]	13	[0.3]	< 0.001
Chronic renal failure	964	[1.3]	958	[1.3]	6	[0.2]	< 0.001
Peripheral arterial disease	291	[0.4]	289	[0.4]	2	[0.1]	< 0.001
Active cancer/chemotherapy	593	[0.8]	583	[0.8]	10	[0.3]	< 0.001
Dementia	2820	[3.7]	2808	[3.9]	12	[0.3]	< 0.001
Substance abuse	15,329	[20.3]	14,525	[20.3]	804	[20.2]	0.912
Any comorbidity	43,524	[57.6]	41,809	[58.4]	1715	[43.1]	< 0.001
Injury characteristics							
AIS head							
3	35,567	[47.1]	34,111	[47.6]	1456	[36.6]	< 0.001
4	25,499	[33.7]	24,155	[33.7]	1344	[33.8]	
5	14,504	[19.2]	13,327	[18.6]	1177	[29.6]	
AIS face = 2	19,676	[26.0]	17,937	[25.1]	1739	[43.7]	< 0.001
AIS neck = 2	650	[0.9]	502	[0.7]	148	[3.7]	< 0.001
AIS chest = 2	7021	[9.3]	6856	[9.6]	165	[4.1]	< 0.001
AIS abdomen = 2	2092	[2.8]	2027	[2.8]	65	[1.6]	< 0.001
AIS spine = 2	10,635	[14.1]	10,479	[14.6]	156	[3.9]	< 0.001
AIS upper extremity = 2	11,574	[15.3]	11,332	[15.8]	242	[6.1]	< 0.001
AIS lower extremity = 2	8720	[11.5]	8654	[12.1]	66	[1.7]	< 0.001
VTE type and timing							
Type of VTE prophylaxis							
UH	30,106	[39.8]	28,613	[40.0]	1493	[37.5]	0.002
LMWH	45,464	[60.2]	42,980	[60.0]	2484	[62.5]	
Time of VTE prophylaxis							
Early prophylaxis (< 48 h)	10,466	[13.8]	9842	[13.7]	624	[15.7]	< 0.001
Procedure							
Cranio-/craniectomy or ICP monitoring	12,767	[16.9]	11,391	[15.9]	1376	[34.6]	< 0.001
Outcomes							
Deep vein thrombosis	1622	[2.1]	1483	[2.1]	139	[3.5]	< 0.001
Pulmonary embolism	469	[0.6]	423	[0.6]	46	[1.2]	< 0.001
Venous embolism	1941	[2.6]	1770	[2.5]	171	[4.3]	< 0.001
ICU admission	58,321	[77.2]	54,808	[76.6]	3513	[88.3]	< 0.001
ICU days^a,c^	3	[1–8]	3	[1–7]	5	[2–12]	< 0.001
HLOS (days)^a^	8	[5–15]	8	[5–15]	11	[6–22]	< 0.001
In-hospital mortality	2867	[3.8]	2622	[3.7]	245	[6.2]	< 0.001

Values are numbers [percentages] unless indicated otherwise

*SBP* systolic blood pressure; *HR* heart rate; *GCS* Glasgow coma scale; *chemo* chemotherapy; *AIS* abbreviated injury scale; *VTE* venous thromboembolism; *UH* unfractionated heparin; *LMWH* low molecular weight heparin; *ICP* intracranial pressure; *HLOS* hospital length of stay; *h* hours

^a^Reported as median and interquartile range (IQR)

^b^Includes 2451 (3.2%) missing values

^c^Only for those admitted to ICU

### Independent risk factors for VTE complications

To assess independent predictors for thromboembolic complications multivariable analysis was performed (Supplemental Table 1).

**Fig. 2:**
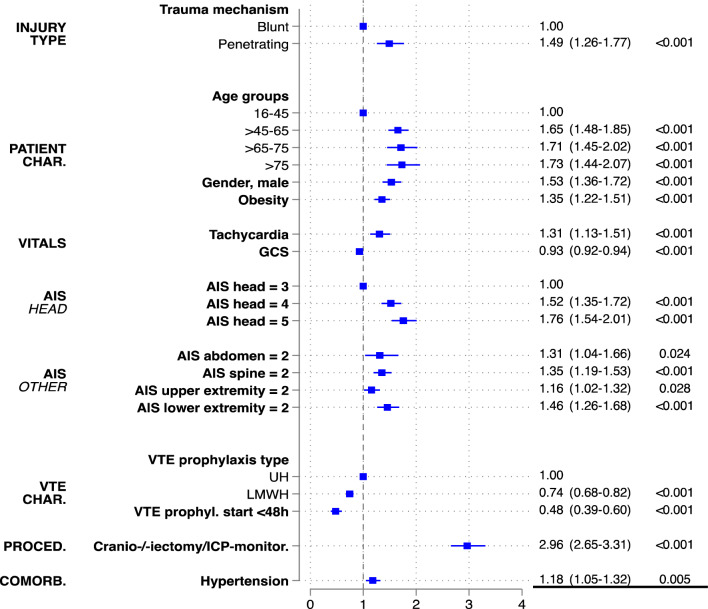
Backward stepwise selection model showing independent predictors for thromboembolic events (Deep vein thrombosis + pulmonary embolism). AUROC = 0.765. *GCS* Glasgow coma scale; *AIS* abbreviated injury scale; VTE venous thromboembolism; *UH* unfractionated heparin; *LMWH* low molecular weight heparin; *ICP* intracranial pressure

Figure 2 shows the final model after stepwise backward selection of important variables predicting VTE events in isolated severe TBI. Penetrating trauma mechanism (OR 1.49, CI 95% 1.26–1.77), increasing age (age 16–45: reference; age > 45–65: OR 1.65, CI 95% 1.48–1.85; age > 65–75: OR 1.71, CI 95% 1.45–2.02; age > 75: OR 1.73, CI 95% 1.44–2.07), male gender (OR 1.53, CI 95% 1.36–1.72), obesity (OR 1.35, CI 95% 1.22–1.51), tachycardia (OR 1.31, CI 95% 1.13–1.51), increasing head AIS (AIS 3: reference; AIS 4: OR 1.52, CI 95% 1.35–1.72; AIS 5: OR 1.76, CI 95% 1.54–2.01), associated moderate injuries (AIS = 2) of the abdomen (OR 1.31, CI 95% 1.04–1.66), spine (OR 1.35, CI 95% 1.19–1.53), upper extremity (OR 1.16, CI 95% 1.02–1.31), lower extremity (OR 1.46, CI 95% 1.26–1.68), craniectomy/craniotomy or ICP monitoring (OR 2.96, CI 95% 2.65–3.31) and pre-existing hypertension (OR 1.18, CI 95% 1.05–1.32) were identified as independent risk factors for VTE complications in isolated severe head injury. Increasing GCS (OR 0.93, CI 95% 0.92–0.94), early VTE prophylaxis (OR 0.48, CI 95% 0.39–0.60) and LMWH compared to heparin (OR 0.74, CI 95% 0.68–0.82) were identified as protective factors for VTE complications.

Supplemental Table 2 shows the multivariable analysis for DVT, Supplemental Table 3 shows the multivariable analysis for PE. Penetrating mechanism of injury was identified as an independent risk factor for both, DVT (Supplemental Table 2, OR 1.42, CI 95% 1.17–1.72) and PE (Supplemental Table 3, OR 1.70, CI 95% 1.22–2.37).

### Independent risk factors for mortality

Furthermore, penetrating trauma was independently associated with higher mortality (OR 1.65, CI 95% 1.40–1.93). An independent trend toward and increased mortality was also observed for pulmonary embolism (OR 1.35 CI 95% 0.97–1.87, *p* = 0.071) (Table 2).

**Table 2:** Multivariable analysis showing independent risk factors for mortality

In-hospital mortality	OR	(95% CI)	*p* value
Mechanism of injury			
Blunt	1.00		Reference
Penetrating	1.65	(1.40–1.93)	< 0.001
Age			
16–45	1.00		Reference
> 45–65	2.61	(2.33–2.91)	< 0.001
> 65–75	4.50	(3.90–5.20)	< 0.001
> 75	8.38	(7.20–9.74)	< 0.001
Gender, male	1.34	(1.22–1.47)	< 0.001
Obesity (BMI > 30 kg/m^2^)	1.08	(0.98–1.20)	0.114
Race			
White	1.00		Reference
Black	0.84	(0.74–0.95)	0.004
Asian	0.93	(0.75–1.15)	0.523
Other	0.94	(0.83–1.06)	0.331
Tachycardia (> 120 bpm)	1.36	(1.20–1.55)	< 0.001
Hypotension [SBP < 120 mmHg]	2.22	(1.79–2.75)	< 0.001
GCS	0.85	(0.84–0.86)	< 0.001
Comorbidities			
Steroid use	1.40	(0.87–2.27)	0.168
Current smoker	0.78	(0.68–0.89)	< 0.001
Diabetes mellitus	1.15	(1.03–1.30)	0.017
Hypertension	1.03	(0.93–1.13)	0.618
Cerebrovascular accident	1.10	(0.87–1.39)	0.416
Respiratory disease	1.53	(1.29–1.80)	< 0.001
Congestive heart failure	1.11	(0.88–1.40)	0.357
Myocardial infarction (past)	1.41	(0.96–2.08)	0.083
Liver cirrhosis	2.92	(2.28–3.75)	< 0.001
Chronic renal failure	2.14	(1.67–2.74)	< 0.001
Peripheral arterial disease	1.50	(0.95–2.36)	0.084
Active cancer/chemotherapy	2.12	(1.55–2.90)	< 0.001
Dementia	1.04	(0.86–1.27)	0.654
Substance abuse disorder	0.96	(0.86–1.07)	0.439
AIS head			
3	1.00		Reference
4	1.55	(1.37–1.75)	< 0.001
5	4.25	(3.78–4.79)	< 0.001
AIS face = 2	0.99	(0.90–1.09)	0.843
AIS neck = 2	1.02	(0.67–1.56)	0.929
AIS chest = 2	1.14	(0.99–1.31)	0.062
AIS abdomen = 2	1.28	(1.01–1.61)	0.039
AIS spine = 2	1.19	(1.06–1.34)	0.004
AIS upper extremity = 2	0.94	(0.82–1.07)	0.328
AIS lower extremity = 2	1.04	(0.90–1.21)	0.587
VTE prophylaxis type			
UH	1.00		Reference
LMWH	0.64	(0.59–0.70)	< 0.001
Early VTE prophylaxis (< 48 h)	0.96	(0.82–1.12)	0.604
Cranio-/craniectomy or ICP monitoring	2.41	(2.19–2.64)	< 0.001
Pulmonary embolism	1.35	(0.97–1.87)	0.071

In total were 75,570 observations included in the final model. Multicollinearity test was checked before doing multivariate analysis. AUROC = 0.866

*SBP* systolic blood pressure; *HR* heart rate; *GCS* Glasgow coma scale; *AIS* abbreviated injury scale; *VTE* venous thromboembolism; *UH* unfractionated heparin; *LMWH* low molecular weight heparin; *ICP* intracranial pressure; *HLOS* hospital length of stay; *h* hours

^a^AIS external includes AIS other and unspecified

### Sensitivity analysis

In addition, a 1:4 case control matching of patients with penetrating vs blunt isolated severe TBI was performed on the basis of the following criteria: age, sex, head AIS, extracranial AIS 2 for face, neck, chest, abdomen, spine, upper and lower extremities. The subsequent regression analysis to identify risk factors for VTE events generated similar results as our previous stepwise backward selection model in both magnitude and direction (Supplemental Table 4).

## Discussion

VTE events in TBI remain a major problem with a significant impact on complication rates, and mortality. A recently published systematic review showed that 20% of patients with isolated TBI had laboratory coagulopathy on hospital admission [16]. However, the effect of TBI on coagulation is not well understood, and in particular, the role of the mechanism of injury with respect to VTE events has not been clinically determined.

Our hypothesis that penetrating compared to blunt trauma mechanism is independently associated with more VTE events in TBI was confirmed. This is an interesting finding that, to our knowledge, has not been described previously.

To understand the role of the mechanism of injury in VTE complications, it is important to be familiar with the underlying interactions of the coagulation cascade that may contribute to VTE complications in trauma and particularly in TBI. In an early phase after trauma underlying mechanisms such as platelet dysfunction [17], hypoperfusion induced activation of protein C may contribute to a hypocoagulable state [18]. In the further course, hypocoagulability seems to transform to a hypercoagulable state with an increased risk of thrombus formation. In this phase the release of tissue factors from local tissue injury, as well as increased systemic production may activate the extrinsic clotting cascade [19, 20]. These findings support the clinical hypothesis that tissue factors drive hypercoagulability after brain injury. Another study by Meizoso et al. [21] recently measured thrombelastography indices in trauma patients with and without TBI. The study provided evidence that fibrinolysis shutdown associated with a hypercoagulable state was more common in patients with TBI as compared to patients with no TBI (25% vs 18%, *p* < 0.0001). Finally, it is discussed that in shock, massive consumption of protein C and Interleukin 6 secretion may also play a role in thrombus formation [22, 23]. Only one study by Martin et al. [11] compared blunt vs penetrating TBI patients with regard to coagulopathy [measured by thromboelastography (TEG) and need for transfusion]. Patients with penetrating head injuries were more coagulopathic by TEG and were also more likely to undergo transfusion with any type of blood product compared to those with blunt injuries (26.5% vs 6%, *p* < 0.0001). Unfortunately, no VTE complications were reported in this study. Nevertheless, it is likely that the group of patients with penetrating injuries who initially presented with accentuated coagulopathy compared to blunt trauma patients, may also have experienced an increased prothrombotic state during the clinical course.

Most clinical research on VTE complications in TBI has focused on blunt trauma. Only a limited number of studies included patients with penetrating injuries. In a retrospective database review of a general trauma population, rates of VTE were the same for blunt and penetrating mechanism [24]. However, in another study from 2021 [25] firearm injury was identified as the strongest mechanism of injury associated with VTE complications in a general trauma population (OR 1.88, CI 95% 1.39–2.54; reference: other injury).

Another study by Meyer et al. [26] was evaluating VTE chemoprophylaxis in penetrating TBI. Despite very early VTE chemoprophylaxis (within 24 h) the study reported VTE rates of 12% in combat-related penetrating brain injury. This high rate may support our findings identifying penetrating injury mechanism as an independent risk factor for VTE complications in TBI.

The decision to start VTE prophylaxis in TBI is extremely challenging: the initiation of VTE prophylaxis too late may result in thrombosis; whereas the initiation of premature prophylaxis may increase the risk of progression of intracranial bleeding, especially in severe TBI [27]. The American College of Surgeons state in its best practice guidelines for the management of TBI that in most cases, VTE prophylaxis should be considered within the first 72 h after TBI [28]. However, several recently published studies evaluated a more aggressive VTE prophylaxis in TBI patients and concluded that VTE prophylaxis within 48 h of admission is safe and effective in preventing VTE complications in TBI patients [29–33]. As a consequence of the increased VTE risk in penetrating as compared to blunt TBI an even more aggressive VTE prophylaxis might be appropriate. In the study by Meyer et al. [26] evaluating penetrating TBI the very early administration of VTE prophylaxis (within 24 h) was safe with regard to the progression of intracranial hemorrhage. However, more high quality data are needed to provide sufficient evidence to support this aggressive VTE prophylaxis management in penetrating TBI.

The underlying mechanisms for higher rates of VTE events in penetrating compared to blunt TBI in our study could not be examined. Higher release of procoagulants (tissue factors) or an increased fibrinolysis shutdown in penetrating compared to blunt trauma would be a possible explanation but is only hypothesized [10]. Further studies should focus on a better understanding of the effects of the mechanism of injury on the coagulation cascade in TBI. This may help to further improve risk stratification regarding the initiation of VTE prophylaxis.

In addition to the penetrating mechanism of injury, the present study identified the following risk factors for VTE events in isolated severe TBI: increasing age, male gender, obesity, tachycardia, increasing head AIS, associated moderate abdominal, spinal, upper or lower extremity injuries defined as AIS 2, neurosurgical procedures (craniectomy/craniotomy or ICP monitoring) and pre-existing hypertension. Increasing GCS, early VTE prophylaxis and LMWH compared to heparin were identified as protective factors.

A study from 2019 using a national registry also reported on risk factors for VTE following TBI [34]. This study retrospectively evaluated 424,929 patients with TBI between 2002 and 2014. The overall described VTE rate was 3.9% compared to 2.6% reported in our study. The higher VTE rate compared to our study may be explained by the fact that patients who died within 72 h were not excluded in this study. These are likely the most severely injured patients with the highest risk for VTE complications. In addition, the study included patients with severe associated injuries which also may have contributed to higher VTE rates. However, in line with our findings the study identified older age, hypertension, obesity, neurosurgical procedures (craniotomy/craniectomy, EVD or ICP monitor) and more severe TBI as independent risk factors for VTE complications. In comparison to our study which showed an independent trend toward an increased mortality for pulmonary embolism [1.35 (95% CI = 0.97–1.87)], the study by Hoffman et al. identified VTE as a protective factor for mortality [0.53 (95% CI = 0.50–0.57)]. This counterintuitive finding may be explained by confounding factors that were not considered in the study design; or may also be explained by the fact that patients who died had a shorter length of stay and therefore had less time to develop a DVT or PE. The following limitations of the study by Hoffman et al. must be taken into account: First, associated injuries were neither reported nor included in the regression analysis. Our study showed that even moderate extracranial injuries (AIS = 2) of the abdomen, spine, and lower/ upper extremities were independently associated with VTE complications in patients with TBI. Second, duration of consciousness was used as a surrogate measure of the severity of TBI because GCS was not coded in the database. However, unconsciousness, for example, is also induced by severe hemorrhagic shock and is therefore an unreliable method for assessing the severity of TBI. Lastly, the study by Hoffman et al. did not consider trauma mechanism (blunt vs penetrating), VTE type and timing in the regression analysis. Not taking these factors into account including the inaccurate surrogate marker for severity of TBI may have confounded the results.

Another large retrospective database study using the National Inpatient Sample database evaluated in 2022 risk factors for VTE complications in adult patients with severe TBI [35]. This study evaluated 349,165 TBI hospitalizations. In line with our findings, the study found an independent association between craniectomy and an increased risk of VTE for patients with severe TBI (OR 1.29, *p* < 0.005). Furthermore age (OR 1.26, *p* < 0.005), chronic lung disease (OR 1.58, *p* < 0.05), electrolyte imbalance (OR 1.43, *p* < 0.05), liver disease (OR 0.10, *p* < 0.05), urinary tract infection (OR 1.56, *p* < 0.05), pneumonia (OR, 2.03, *p* < 0.0001), and sepsis (OR 1.57, *p* < 0.05) were also identified as independent factors associated with VTE events. Obesity (OR, 2.09, *p* > 0.05) and spine injury (OR 2.03; *p* > 0.05) showed a trend toward an increased independent risk for VTE events. In this study, associated severe injuries were also not excluded, and the regression analysis corrected only for spinal injuries and leg or pelvic fractures. On the other hand, complications such as sepsis, pneumonia, urinary tract infections, acute myocardial infarction, or cardiac arrest were included in the regression analysis. In our opinion, the inclusion of complications as predictive variables for VTE events is of limited use, because the mentioned complications develop during the clinical course and hardly contribute to the necessary early risk stratification for thromboembolic events.

### Strengths and limitations

To our knowledge, this is the first study identifying penetrating trauma mechanism as an independent risk factor for VTE events in isolated severe TBI. Definitely a strength of this study includes the large number of patients, the quality of the TQIP databank, and the evaluation of patients with isolated severe TBI. This helps minimize confounding effects of severe non-TBI injuries with regard to VTE events. In addition, to assess a homogenous population, only patients receiving VTE prophylaxis with either unfractionated heparin or low-molecular-weight heparin were included in the analysis. Furthermore, a broad number of potential confounders were considered for analyses and the risk factors for VTE events in isolated severe head injury were tested in an additional case control matching analysis.

However, there are a number of limitations and our results should be interpreted with caution. First this is a retrospective study, based on a large database and it is therefore associated with all the inherent limitations of this study design. The strength to evaluate only patients with isolated severe TBI is also a limitation because patients with TBI often present with severe concomitant injuries. As our results showed, even moderate associated injuries (AIS = 2) increased the risk of VTE complications. Therefore, it is very likely that VTE rates are even higher in polytraumatized patients with combined head and associated injuries. In addition, doses, duration and held doses after initiation of VTE prophylaxis, including clotting tests, are not recorded in the TQIP database and could not be considered for analysis. Finally, the present study did not examine specific mechanisms of injury (gunshot or stab wound for penetrating injury). This could be the focus of future studies.

## Conclusions

Penetrating injury mechanism, increasing age, male gender, obesity, tachycardia, increasing head AIS, associated moderate injuries (AIS = 2) of the abdomen, spine, upper or lower extremity, neurosurgical procedures (craniectomy/craniotomy or ICP monitoring) and pre-existing hypertension were associated with an increased VTE risk in isolated severe TBI. Increasing GCS, early VTE prophylaxis and LMWH compared to heparin were identified as protective factors. The identified factors associated with VTE complications will help for improved risk stratification. In penetrating compared to blunt TBI an even more aggressive VTE prophylaxis management may be appropriate.

### Supplementary Information

Below is the link to the electronic supplementary material.

Supplemental Table 1 Multivariable analysis showing independent risk factors for venous thromboembolsim (Deep vein thrombosis + pulmonary embolism). Supplementary file1 (DOCX 19 KB)

Supplemental Table 2 Multivariable analysis showing independent risk factors for deep vein thrombosis. Supplementary file2 (DOCX 19 KB)

Supplemental Table 3 Multivariable analysis showing independent risk factors for pulmonary embolism. Supplementary file3 (DOCX 19 KB)

Supplemental Table 4 Adjusted effects of risk factors for venous thromboembolism after case control matching adjusted on age, sex, head AIS, extracranial AIS 2 for face, neck, chest abdomen, spine, upper and lower extremity. Supplementary file4 (DOCX 15 KB)
