# Supplementary material for: Risk factors for thromboembolic complications in isolated severe head injury
Source: Eur J Trauma Emerg Surg. 2023 Jun 8;50(1):185–95. doi: 10.1007/s00068-023-02292-y (PMC10923954; doi:10.1007/s00068-023-02292-y)
Supplement: Supplementary file 1 — Multivariable analysis showing independent risk factors for venous thromboembolsim (Deep vein thrombosis + pulmonary embolism). Supplementary file1 (DOCX 19 KB) [file 68_2023_2292_MOESM1_ESM.docx]

| ***Venous Thromboembolism*** |  | **OR** |  | **(95% CI)** |  | **p-value** |
| --- | --- | --- | --- | --- | --- | --- |
| **Mechanism of injury** |  | |  | |  | |
| Blunt | 1.00 | |  | | *reference* | |
| Penetrating | 1.48 | | (1.24-1.77) | | <0.001 | |
|  |  | |  | |  | |
| **Age** |  | |  | |  | |
| 16-45 | 1.00 | |  | | *reference* | |
| >45-65 | 1.65 | | (1.47-1.85) | | <0.001 | |
| >65-75 | 1.73 | | (1.46-2.05) | | <0.001 | |
| >75 | 1.80 | | (1.49-2.18) | | <0.001 | |
|  |  | |  | |  | |
| **Gender, male** | 1.57 | | (1.40-1.76) | | <0.001 | |
|  |  | |  | |  | |
| **Obesity (BMI >30kg/m2)** | 1.36 | | (1.22-1.51) | | <0.001 | |
|  |  | |  | |  | |
| **Race** |  | |  | |  | |
| White | 1.00 | |  | | *reference* | |
| Black | 1.04 | | (0.92-1.18) | | 0.513 | |
| Asian | 0.94 | | (0.71-1.23) | | 0.630 | |
| Other | 1.02 | | (0.89-1.17) | | 0.742 | |
|  |  | |  | |  | |
| **Tachycardia (>120bpm)** | 1.30 | | (1.12-1.50) | | <0.001 | |
| **Hypotension[SBP<120mmHg]** | 1.12 | | (0.83; 1.52) | | 0.460 | |
| **GCS** | 0.93 | | (0.92-0.94) | | <0.001 | |
|  |  | |  | |  | |
| **Comorbidities** |  | |  | |  | |
| Steroid use | 1.00 | | (0.49-2.04) | | 0.991 | |
| Current Smoker | 1.00 | | (0.87-1.14) | | 0.997 | |
| Diabetes mellitus | 1.00 | | (0.86-1.17) | | 0.960 | |
| Hypertension | 1.16 | | (1.03-1.31) | | 0.017 | |
| Cerebrovascular Accident | 1.21 | | (0.90-1.63) | | 0.204 | |
| Respiratory disease | 1.03 | | (0.82-1.30) | | 0.771 | |
| Congestive heart failure | 1.24 | | (0.91-1.69) | | 0.179 | |
| Myocardial infarction (past) | 1.51 | | (0.94-2.44) | | 0.088 | |
| Liver cirrhosis | 0.66 | | (0.42-1.06) | | 0.084 | |
| Chronic renal failure | 0.94 | | (0.62-1.42) | | 0.753 | |
| Peripheral Arterial Disease | 0.81 | | (0.36-1.85) | | 0.620 | |
| Active Cancer/ Chemotherapy | 1.11 | | (0.66-1.88) | | 0.699 | |
| Dementia | 0.73 | | (0.53-1.01) | | 0.060 | |
| Substance abuse disorder | 1.09 | | (0.97-1.23) | | 0.148 | |
|  |  | |  | |  | |
| **AIS head** |  | |  | |  | |
| 3 | 1.00 | |  | | *reference* | |
| 4 | 1.52 | | (1.34-1.72) | | <0.001 | |
| 5 | 1.75 | | (1.53-2.00) | | <0.001 | |
|  |  | |  | |  | |
| **AIS face** **=** **2** | 0.96 | | (0.86-1.07) | | 0.479 | |
|  |  | |  | |  | |
| **AIS neck** **=** **2** | 1.16 | | (0.75-1.79) | | 0.497 | |
|  |  | |  | |  | |
| **AIS chest** **=** **2** | 1.04 | | (0.90-1.22) | | 0.576 | |
|  |  | |  | |  | |
| **AIS abdomen** **=** **2** | 1.30 | | (1.02-1.65) | | 0.031 | |
|  |  | |  | |  | |
| **AIS spine** **=** **2** | 1.34 | | (1.18-1.53) | | <0.001 | |
|  |  | |  | |  | |
| **AIS upper extremity** **=** **2** | 1.16 | | (1.02-1.32) | | 0.028 | |
|  |  | |  | |  | |
| **AIS lower extremity** **=** **2** | 1.45 | | (1.26-1.68) | | <0.001 | |
|  |  | |  | |  | |
| **VTE prophylaxis type** |  | |  | |  | |
| UH | 1.00 | |  | | *reference* | |
| LMWH | 0.75 | | (0.68-0.82) | | <0.001 | |
|  |  | |  | |  | |
| **Early VTE prophylaxis (<48h)** | 0.48 | | (0.39-0.59) | | <0.001 | |
|  |  | |  | |  | |
| **Cranio-/Craniectomy or ICP** | 2.98 | | (2.66-3.32) | | <0.001 | |
| **monitoring** |  | |  | |  | |
