# Supplementary material for: Risk factors for thromboembolic complications in isolated severe head injury
Source: Eur J Trauma Emerg Surg. 2023 Jun 8;50(1):185–95. doi: 10.1007/s00068-023-02292-y (PMC10923954; doi:10.1007/s00068-023-02292-y)
Supplement: Supplementary file 2 — Multivariable analysis showing independent risk factors for deep vein thrombosis. Supplementary file2 (DOCX 19 KB) [file 68_2023_2292_MOESM2_ESM.docx]

| ***Deep Vein Thrombosis*** |  | **OR** |  | **(95% CI)** |  | **p-value** |
| --- | --- | --- | --- | --- | --- | --- |
| **Mechanism of injury** |  | |  | |  | |
| Blunt | 1.00 | |  | | *reference* | |
| Penetrating | 1.42 | | (1.17-1.72) | | <0.001 | |
|  |  | |  | |  | |
| **Age** |  | |  | |  | |
| 16-45 | 1.00 | |  | | *reference* | |
| >45-65 | 1.70 | | (1.50-1.92) | | <0.001 | |
| >65-75 | 1.77 | | (1.48-2.13) | | <0.001 | |
| >75 | 1.76 | | (1.42-2.17) | | <0.001 | |
|  |  | |  | |  | |
| **Gender, male** | 1.49 | | (1.31-1.69) | | <0.001 | |
|  |  | |  | |  | |
| **Obesity (BMI >30kg/m2)** | 1.42 | | (1.26-1.59) | | <0.001 | |
|  |  | |  | |  | |
| **Race** |  | |  | |  | |
| White | 1.00 | |  | | *reference* | |
| Black | 1.02 | | (0.89-1.17) | | 0.783 | |
| Asian | 0.96 | | (0.72-1.29) | | 0.809 | |
| Other | 1.04 | | (0.90-1.20) | | 0.614 | |
|  |  | |  | |  | |
| **Tachycardia (>120bpm)** | 1.27 | | (1.08-1.49) | | 0.003 | |
| **Hypotension[SBP<120mmHg]** | 1.11 | | (0.79-1.55) | | 0.545 | |
| **GCS** | 0.93 | | (0.92-0.94) | | <0.001 | |
|  |  | |  | |  | |
| **Comorbidities** |  | |  | |  | |
| Steroid use | 1.04 | | (0.48-2.23) | | 0.926 | |
| Current Smoker | 0.98 | | (0.85-1.14) | | 0.796 | |
| Diabetes mellitus | 1.07 | | (0.91-1.26) | | 0.424 | |
| Hypertension | 1.11 | | (0.97-1.26) | | 0.122 | |
| Cerebrovascular Accident | 1.31 | | (0.96-1.78) | | 0.092 | |
| Respiratory disease | 0.98 | | (0.76-1.27) | | 0.882 | |
| Congestive heart failure | 1.31 | | (0.94-1.84) | | 0.109 | |
| Myocardial infarction (past) | 1.40 | | (0.82-2.38) | | 0.216 | |
| Liver cirrhosis | 0.74 | | (0.46-1.19) | | 0.213 | |
| Chronic renal failure | 0.98 | | (0.63-1.52) | | 0.916 | |
| Peripheral Arterial Disease | 0.48 | | (0.15-1.51) | | 0.208 | |
| Active Cancer/ Chemotherapy | 1.25 | | (0.72-2.15) | | 0.426 | |
| Dementia | 0.79 | | (0.56-1.12) | | 0.192 | |
| Substance abuse disorder | 1.12 | | (0.99-1.27) | | 0.083 | |
|  |  | |  | |  | |
| **AIS head** |  | |  | |  | |
| 3 | 1.00 | |  | | *reference* | |
| 4 | 1.55 | | (1.35-1.77) | | <0.001 | |
| 5 | 1.84 | | (1.59-2.13) | | <0.001 | |
|  |  | |  | |  | |
| **AIS face=2** | 0.94 | | (0.83-1.05) | | 0.273 | |
|  |  | |  | |  | |
| **AIS neck=2** | 1.12 | | (0.69-1.81) | | 0.659 | |
|  |  | |  | |  | |
| **AIS chest=2** | 0.98 | | (0.82-1.16) | | 0.795 | |
|  |  | |  | |  | |
| **AIS abdomen=2** | 1.16 | | (0.89-1.53) | | 0.278 | |
|  |  | |  | |  | |
| **AIS spine=2** | 1.29 | | (1.12-1.49) | | <0.001 | |
|  |  | |  | |  | |
| **AIS upper extremity=2** | 1.14 | | (0.99-1.32) | | 0.073 | |
|  |  | |  | |  | |
| **AIS lower extremity=2** | 1.49 | | (1.27-1.74) | | <0.001 | |
|  |  | |  | |  | |
| **VTE prophylaxis type** |  | |  | |  | |
| UH | 1.00 | |  | | *reference* | |
| LMWH | 0.78 | | (0.70-0.86) | | <0.001 | |
|  |  | |  | |  | |
| **Early VTE prophylaxis (<48h)** | 0.49 | | (0.39-0.62) | | <0.001 | |
|  |  | |  | |  | |
| **Cranio-/Craniectomy or ICP** | 3.07 | | (2.72-3.46) | | <0.001 | |
| **monitoring** |  | |  | |  | |
