# Supplementary material for: Risk factors for thromboembolic complications in isolated severe head injury
Source: Eur J Trauma Emerg Surg. 2023 Jun 8;50(1):185–95. doi: 10.1007/s00068-023-02292-y (PMC10923954; doi:10.1007/s00068-023-02292-y)
Supplement: Supplementary file 3 — Multivariable analysis showing independent risk factors for pulmonary embolism. Supplementary file3 (DOCX 19 KB) [file 68_2023_2292_MOESM3_ESM.docx]

| ***Pulmonary Embolism*** |  | **OR** |  | **(95% CI)** |  | **p-value** |
| --- | --- | --- | --- | --- | --- | --- |
| **Mechanism of injury** |  | |  | |  | |
| Blunt | 1.00 | |  | | *reference* | |
| Penetrating | 1.70 | | (1.22-2.37) | | 0.002 | |
|  |  | |  | |  | |
| **Age** |  | |  | |  | |
| 16-45 | 1.00 | |  | | *reference* | |
| >45-65 | 1.60 | | (1.27-2.00) | | <0.001 | |
| >65-75 | 1.40 | | (0.98-2.00) | | 0.064 | |
| >75 | 1.94 | | (1.33-2.81) | | <0.001 | |
|  |  | |  | |  | |
| **Gender, male** | 1.61 | | (1.27-2.04) | | <0.001 | |
|  |  | |  | |  | |
| **Obesity (BMI >30kg/m2)** | 1.21 | | (0.97-1.51) | | 0.086 | |
|  |  | |  | |  | |
| **Race** |  | |  | |  | |
| White | 1.00 | |  | | *reference* | |
| Black | 1.05 | | (0.82-1.35) | | 0.685 | |
| Asian | 0.88 | | (0.50-1.55) | | 0.659 | |
| Other | 0.99 | | (0.76-1.30) | | 0.955 | |
|  |  | |  | |  | |
| **Tachycardia (>120bpm)** | 1.43 | | (1.09-1.88) | | 0.010 | |
| **Hypotension[SBP<120mmHg]** | 1.55 | | (0.94-2.56) | | 0.086 | |
| **GCS** | 0.93 | | (0.91-0.96) | | <0.001 | |
|  |  | |  | |  | |
| **Comorbidities** |  | |  | |  | |
| Steroid use | 0.55 | | (0.08-3.94) | | 0.550 | |
| Current Smoker | 1.12 | | (0.86-1.45) | | 0.402 | |
| Diabetes mellitus | 0.86 | | (0.63-1.18) | | 0.359 | |
| Hypertension | 1.35 | | (1.06-1.72) | | 0.014 | |
| Cerebrovascular Accident | 1.17 | | (0.63-2.15) | | 0.625 | |
| Respiratory disease | 1.08 | | (0.69-1.70) | | 0.732 | |
| Congestive heart failure | 0.99 | | (0.50-1.97) | | 0.972 | |
| Myocardial infarction (past) | 1.67 | | (0.68-4.14) | | 0.264 | |
| Liver cirrhosis | 0.15 | | (0.02-1.11) | | 0.063 | |
| Chronic renal failure | 0.48 | | (0.15-1.52) | | 0.210 | |
| Peripheral Arterial Disease | 1.82 | | (0.57-5.82) | | 0.311 | |
| Active Cancer/ Chemotherapy | 0.63 | | (0.16-2.55) | | 0.516 | |
| Dementia | 0.48 | | (0.22-1.04) | | 0.064 | |
| Substance abuse disorder | 1.00 | | (0.79-1.27) | | 0.992 | |
|  |  | |  | |  | |
| **AIS head** |  | |  | |  | |
| 3 | 1.00 | |  | | *reference* | |
| 4 | 1.43 | | (1.13-1.82) | | 0.003 | |
| 5 | 1.42 | | (1.09-1.86) | | 0.009 | |
|  |  | |  | |  | |
| **AIS face=2** | 1.13 | | (0.92-1.39) | | 0.254 | |
|  |  | |  | |  | |
| **AIS neck=2** | 1.08 | | (0.47-2.45) | | 0.860 | |
|  |  | |  | |  | |
| **AIS chest=2** | 1.25 | | (0.94-1.65) | | 0.122 | |
|  |  | |  | |  | |
| **AIS abdomen=2** | 1.70 | | (1.14-2.55) | | 0.010 | |
|  |  | |  | |  | |
| **AIS spine=2** | 1.64 | | (1.29-2.08) | | <0.001 | |
|  |  | |  | |  | |
| **AIS upper extremity=2** | 1.26 | | (0.98-1.61) | | 0.068 | |
|  |  | |  | |  | |
| **AIS lower extremity=2** | 1.25 | | (0.94-1.66) | | 0.120 | |
|  |  | |  | |  | |
| **VTE prophylaxis type** |  | |  | |  | |
| UH | 1.00 | |  | | *reference* | |
| LMWH | 0.59 | | (0.49-0.71) | | <0.001 | |
|  |  | |  | |  | |
| **Early VTE prophylaxis (<48h)** | 0.52 | | (0.34-0.78) | | 0.002 | |
|  |  | |  | |  | |
| **Cranio-/Craniectomy or ICP** | 2.66 | | (2.13-3.32) | | <0.001 | |
| **monitoring** |  | |  | |  | |
