# Supplementary material for: Risk factors for thromboembolic complications in isolated severe head injury
Source: Eur J Trauma Emerg Surg. 2023 Jun 8;50(1):185–95. doi: 10.1007/s00068-023-02292-y (PMC10923954; doi:10.1007/s00068-023-02292-y)
Supplement: Supplementary file 4 — Adjusted effects of risk factors for venous thromboembolism after case control matching adjusted on age, sex, head AIS, extracranial AIS 2 for face, neck, chest abdomen, spine, upper and lower extremity. Supplementary file4 (DOCX 15 KB) [file 68_2023_2292_MOESM4_ESM.docx]

| ***Venous Thromboembolism*** |  | **OR** |  | **(95% CI)** |  | **p-value** |
| --- | --- | --- | --- | --- | --- | --- |
| **Mechanism of injury** |  | |  | |  | |
| Blunt | 1.00 | |  | | *reference* | |
| Penetrating | 1.27 | | (1.03-1.56) | | 0.027 | |
|  |  | |  | |  | |
| **Obesity (BMI >30kg/m2)** | 1.43 | | (1.12-1.82) | | 0.004 | |
|  |  | |  | |  | |
| **Tachycardia (>120bpm)** | 1.67 | | (1.24-2.24) | | 0.001 | |
| **GCS** | 0.92 | | (0.90-0.94) | | <0.001 | |
|  |  | |  | |  | |
| **Comorbidities** |  | |  | |  | |
| Arterial hypertension | 1.29 | | (0.96-1.76) | | 0.094 | |
|  |  | |  | |  | |
| **VTE prophylaxis type** |  | |  | |  | |
| UH | 1.00 | |  | | *reference* | |
| LMWH | 0.66 | | (0.54-0.81) | | <0.001 | |
|  |  | |  | |  | |
| **Early VTE prophylaxis (<48h)** | 0.44 | | (0.27-0.70) | | 0.001 | |
|  |  | |  | |  | |
| **Cranio-/Craniectomy or ICP** | 2.57 | | (2.04-3.23) | | <0.001 | |
| **monitoring** |  | |  | |  | |
